# Deciphering breast cancer dynamics: insights from single-cell and spatial profiling in the multi-omics era

**DOI:** 10.1186/s40364-024-00654-1

**Published:** 2024-09-18

**Authors:** Xin Xiong, Xin Wang, Cui-Cui Liu, Zhi-Ming Shao, Ke-Da Yu

**Affiliations:** 1 Department of Breast Surgery, Key Laboratory of Breast Cancer in Shanghai, Cancer Institute, Fudan University Shanghai Cancer Center, Department of Oncology, Shanghai Medical College, Fudan University, Shanghai, 200032 China; 2 Department of Anesthesiology, Fudan University Shanghai Cancer Center, Shanghai Medical College, Fudan University, Shanghai, 200032 China

**Keywords:** Breast cancer, Single-cell sequencing, Spatial omics, Tumor heterogeneity

## Abstract

As one of the most common tumors in women, the pathogenesis and tumor heterogeneity of breast cancer have long been the focal point of research, with the emergence of tumor metastasis and drug resistance posing persistent clinical challenges. The emergence of single-cell sequencing (SCS) technology has introduced novel approaches for gaining comprehensive insights into the biological behavior of malignant tumors. SCS is a high-throughput technology that has rapidly developed in the past decade, providing high-throughput molecular insights at the individual cell level. Furthermore, the advent of multitemporal point sampling and spatial omics also greatly enhances our understanding of cellular dynamics at both temporal and spatial levels. The paper provides a comprehensive overview of the historical development of SCS, and highlights the most recent advancements in utilizing SCS and spatial omics for breast cancer research. The findings from these studies will serve as valuable references for future advancements in basic research, clinical diagnosis, and treatment of breast cancer.

## Background

Breast cancer is one of the most common malignant tumors in women, and a study by the World Health Organization's International Agency for Research on Cancer shows that there will be 2.3 million new cases of breast cancer worldwide in 2022 and an estimated 666,000 deaths [1]. Breast cancer is a highly heterogeneous disease, based on the presence of hormone receptor (HR) [estrogen receptor (ER) and progesterone receptor (PR) status], human epidermal growth factor receptor 2 (HER2) status, and the proliferation index Ki67, breast cancer can be classified into four different molecular subtypes [2]: Luminal A, Luminal B, HER2 + , and triple-negative breast cancer (TNBC); according to the intrinsic gene expression characteristics, breast cancer can be classified into five molecular subtypes: Luminal A, Luminal B, Normal-like, HER2-enriched, and Basal-like [3]. For treatment purposes, breast cancer is generally divided into three clinical subtypes: HR-positive/HER2-negative (HR + /HER2-), HER2-positive (HER2 +), and TNBC [HR-negative (HR-), HER2-] [4, 5]. All types of tumors, including breast cancer, are intricate and dynamic diseases, progressing from non-malignant to malignant states, from situ lesions to invasive forms, and from sensitivity to resistance to treatment [6]. Researchers have dedicated considerable efforts over the years to address these complexities, aiming to enhance our understanding of tumors and facilitate the development of more precise treatments.

**Fig. 1:**
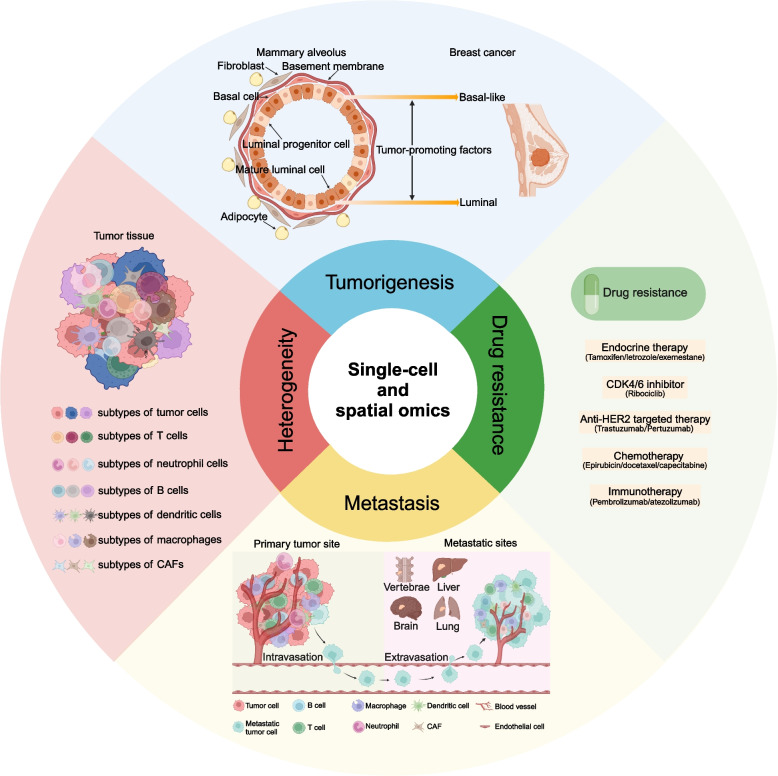
Overview of the application of single-cell and spatial omics in breast cancer. The application of single-cell and spatial omics enables the elucidation of the origin, heterogeneity, and underlying mechanisms governing metastasis and drug resistance in breast cancer. Abbreviations: CAF, Cancer-associated fibroblast

The emergence of traditional high-throughput sequencing technology has promoted the exploration of the mechanism of tumorigenesis and progression and accelerated the development of precision oncology [7, 8]. However, the results of traditional sequencing technologies present the average signal of many mixed cells and cannot specifically analyze the characteristics of a specific cell population, so there are still some limitations [9, 10]. Unlike traditional sequencing technologies, SCS reveals cellular and microenvironmental features at single-cell resolution and has unique advantages in studying tumorigenesis, metastasis, recurrence, and treatment resistance [11]. The spatial localization characteristics of cells within the tumor microenvironment are also crucial for tumor progression. Using spatial sequencing techniques allows for the matching of sequencing data with the spatial positions of cells, thereby diversifying the perspectives of research [12, 13]. The present article provides a comprehensive overview of the current utilization of SCS and spatial omics in breast cancer research, offering valuable references for comprehending the origin, heterogeneity, metastasis, and drug resistance mechanisms associated with this disease (Fig. 1).

## Evolution and basic framework of single-cell sequencing and spatial omics

### Single-cell sequencing

Since the publication of the first single-cell RNA sequencing (scRNA-seq) data in 2009 [14], SCS has developed rapidly over the years. In 2013, SCS was recognized as the Method of the Year by Nature Methods [15]. In 2019, single-cell multi-omics technology received the prestigious title of Method of the Year from Nature Methods [16]. Presently, SCS has found applications in various medical fields such as oncology [11], pharmacy [17], infectious diseases [18], botany [19, 20], and entomology [21, 22]. SCS mainly includes single-cell genomics [23], single-cell transcriptomics [24], single-cell proteomics [25], single-cell epigenomics [26], single-cell metabolomics [27], and single-cell multi-omics [28], etc. There are multiple well-established commercial sequencing platforms accessible to researchers, such as 10X Genomics [29] and BD Rhapsody [30].

**Fig. 2:**
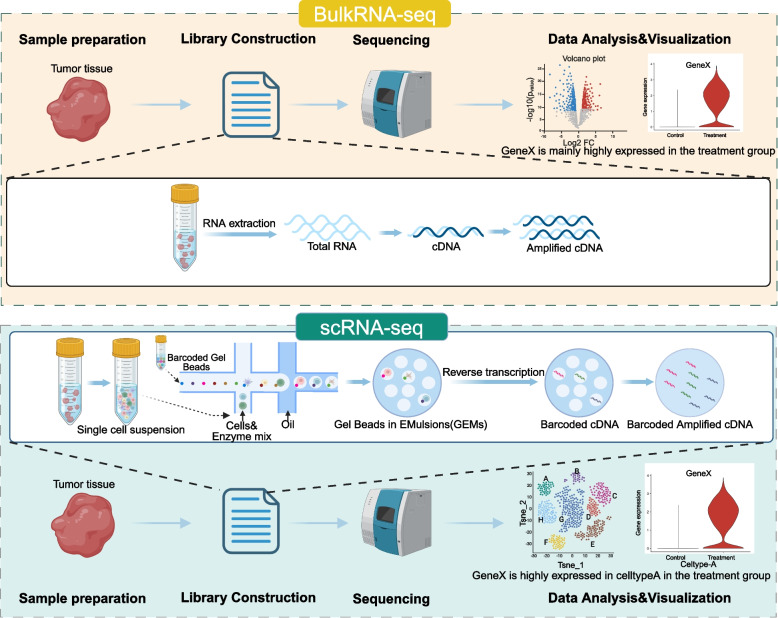
Differences between bulk RNA sequencing and scRNA-seq. Taking RNA sequencing as an example, we can illustrate the distinction between traditional bulk sequencing and SCS (10 × Genomics). Bulk RNA sequencing (Bulk RNA-seq) provides average information obtained from a mixture of cells, commonly utilized for analyzing intergroup differences in gene expression. scRNA-seq dissects tissues into individual cells to capture gene expression profiles at a single-cell level, enabling comparisons of group-specific variations

The scRNA-seq is the most widely used SCS technology. Taking scRNA-seq as an example, the main steps of scRNA-seq encompass tissue dissociation, isolation and capture of individual cells, nucleic acid amplification, library construction, high-throughput sequencing, and data analysis [31, 32]. The isolation and capture of individual cells ensure the acquisition of gene expression information for each cell in subsequent steps, distinguishing scRNA-seq from traditional bulk RNA sequencing (Fig. 2).

Data analysis in scRNA-seq is a complex and time-consuming process that has led to the development of various computational tools for analyzing and visualizing scRNA-seq data [33]. Seurat [34] and scanpy [35] are the primary toolkits used for preliminary processing of scRNA-seq data such as quality control, dimensional reduction clustering, and cell type identification. Additionally, multiple adjunct data analysis processes enable further exploration of single-cell data for more efficient utilization. For instance, Monocle3 is a quasi-temporal analysis toolkit that utilizes scRNA-seq data to infer cell developmental trajectories allowing the discovery of cellular lineage relationships and differentiation status [36]. Cellchat facilitates cell communication analysis by quantitatively describing intercellular communication networks while predicting ligand-receptor interactions between different cells [37]. SCENIC focuses on transcription factors within scRNA-seq datasets to establish gene regulatory networks, providing insights into critical regulators across distinct cell types [38].

### Other single-cell sequencing technologies

The progression from genome to transcriptome, proteome, and ultimately phenotype is a complex, diverse, and non-linear phenomenon [39, 40]. In addition to integrating multiple SCS technologies for comprehensive information acquisition, single-cell multi-omics technologies enable simultaneous retrieval of various types of data in a single sequencing experiment [28]. For instance, the DR-seq (gDNA-mRNA sequencing) enables the simultaneous acquisition of genomic and transcriptomic information [41], and SNARE-seq (single-nucleus chromatin accessibility and mRNA expression sequencing) allows for concurrent profiling of epigenetic and transcriptomic features [42]. The emergence and application of these technologies enable us to extract a greater amount of information from a sample, thereby significantly saving time and enhancing the diversity of obtained information.

Clustered regularly interspaced short palindromic repeats (CRISPR)/CRISPR-associated nuclease 9 (CRISPR/Cas9) genome editing system has been a powerful genome engineering tool, which allows scientists to precisely alter DNA sequences, enabling the observation of cell phenotypes mediated by specific genes [43, 44]. Notably, the single-cell CRISPR screening technology (produced by combining SCS and CRISPR/Cas9 genome editing system) has significantly enhanced the efficacy of gene editing [45]. This advanced technique enables the simultaneous capture of CRISPR single guide RNA and transcriptome information from individual cells, facilitating multi-gene screening within a single experiment and unveiling the potential functional implications of targeted genes [46–48].

### Spatial omics

Although SCS can provide a wealth of information at an exceptionally high resolution, it offers a partial view. For instance, while scRNA-seq enables the identification of distinct cell types, it lacks spatial information about their locations. The emergence of spatial sequencing technologies solves this limitation well.

In 2020, spatially resolved transcriptomics was recognized as the Method of the Year by Nature Methods [49]. The rapid development of spatial omics can be attributed to the advancements in imaging, omics analysis, sequencing, mass spectrometry, image analysis, and bioinformatics [50]. Spatial sequencing technologies use special processing methods to obtain cellular transcriptomic, proteomic, and metabolomic information while preserving the original spatial location of cells in tumor tissue slices [12, 13, 51]. Different spatial sequencing technologies employ distinct methods for acquiring spatial location information. For instance, stereo-seq, a technique for spatial transcriptome sequencing, utilizes in-situ sequencing of spatial barcodes (capture probes—DNA oligonucleotides) to obtain spatial coordinates [52]. On the other hand, CODEX, a technology for spatial protein sequencing, enables the determination of protein locations through multiplexed antibody detection [53]. The processing and analysis of spatial sequencing data are inherently complex, necessitating advanced bioinformatics expertise from researchers [51, 54].

The integration of spatial sequencing data with SCS data is frequently employed in practical applications. By integrating scRNA-seq and spatial transcriptomics data to achieve complementary strengths, gene expression signatures, spatial location localization of cells, and their proximity to each other can be obtained, further deepening our understanding of diseases [55]. Currently, spatial transcriptomics sequencing has been widely used in the study of a variety of tumors, including breast cancer [56], hepatocellular carcinoma [57], colorectal cancer [58], and kidney cancer [59]. Moreover, the combination of high-resolution imaging techniques with mass spectrometry enables spatial proteomics to provide precise information on the distribution and localization of proteins in cells or tissues [60, 61]. The application of spatial proteomics enables the analysis of tumor heterogeneity at the protein level, facilitating the identification of pivotal factors driving tumor progression [62–65]. Metabolic abnormality is a hallmark of tumors, with the most well-known being the Warburg effect [66]. Tumor metabolic characteristics exhibit heterogeneity [67]. Spatial metabolomics is increasingly advancing and enables the assessment of cellular metabolism status in different regions of tumor tissue slices [68, 69], facilitating comprehensive analysis of region-specific metabolic profiles and further exploration into tumor metabolism heterogeneity [70–72].

**Fig. 3:**
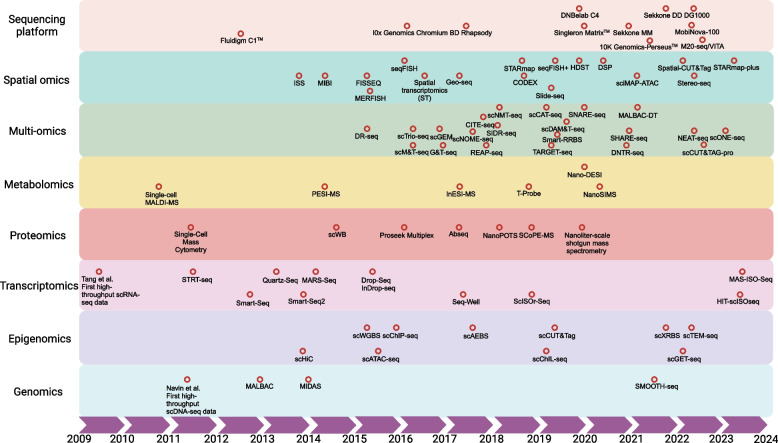
Summary of some important SCS and spatial sequencing technologies and commercial sequencing platforms. Since the pioneering work by Tang et al. in 2009, SCS has witnessed rapid advancements encompassing genomics, transcriptomics, proteomics, epigenomics, metabolomics, spatial omics, and multi-omics. Furthermore, a plethora of well-established commercial platforms have emerged to cater to the diverse needs of researchers. The scatterplot depicted in this diagram illustrates the chronological order of their release dates. The release timing of the commercial platform is contingent upon its public listing schedule

Overall, with the advancement of sequencing technology, an increasing number of SCS and spatial sequencing technologies and commercial platforms are gradually being introduced, enabling researchers to select one or multiple sequencing technologies/commercial platforms based on their research objectives [14, 41, 42, 52, 53, 73–142] (Fig. 3). In addition, the establishment of public databases has significantly enhanced the popularity of SCS technology and spatial omics, enabling multiple databases to be utilized for data acquisition or analysis in the field of SCS and spatial omics [143–164] (Table 1).

**Table 1:** Public databases for single-cell and spatial omics in the medical field

Database	Availability	Function	References
GEO	https://www.ncbi.nlm.nih.gov/geo/	Data download	[143]
The Human Cell Atlas	https://data.humancellatlas.org/	Data download	[144]
CellMarker 2.0	http://bio-bigdata.hrbmu.edu.cn/CellMarker/	Data analysis	[145]
ColorCells	https://rna.sysu.edu.cn/colorcells/index.php	Data analysis	[146]
Human Cell Landscape	https://bis.zju.edu.cn/HCL/	Data analysis	[147]
Mouse Cell Atlas	https://bis.zju.edu.cn/MCA/index.html	Data analysis	[148]
scTPA	http://sctpa.bio-data.cn/sctpa/	Data analysis	[149]
TISCH	http://tisch.comp-genomics.org/home/	Data analysis	[150]
SPDB	https://scproteomicsdb.com/	Data analysis	[151]
SODB	https://gene.ai.tencent.com/SpatialOmics/	Data analysis	[152]
BloodSpot	https://www.fobinf.com/	Data analysis	[153]
SPASCER	https://ccsm.uth.edu/SPASCER/	Data download/Data analysis	[154]
SCPortalen	https://single-cell.riken.jp/SCPortalen_Database/	Data download/Data analysis	[155]
SpatialDB	https://www.spatialomics.org/SpatialDB	Data download/Data analysis	[156]
STOmicsDB	https://db.cngb.org/stomics/	Data download/Data analysis	[157]
Tabula Muris	https://tabula-muris.ds.czbiohub.org/	Data download/Data analysis	[158]
PanglaoDB	https://panglaodb.se/	Data download/Data analysis	[159]
Jinglebells	https://jinglebells.bgu.ac.il/	Data download/Data analysis	[160]
Cell BLAST	https://cblast.gao-lab.org/	Data download/Data analysis	[161]
DISCO	https://www.immunesinglecell.org/	Data download/Data analysis	[162]
CancerSEA	http://biocc.hrbmu.edu.cn/CancerSEA/	Data download/Data analysis	[163]
SC2disease	http://easybioai.com/sc2disease/	Data download/Data analysis	[164]

#### Single-cell sequencing and spatial omics reveal the origin of breast cancer

Breast cancer originates in breast epithelial cells, and the origin cells of different breast cancer subtypes may be different [165]. Lim E et al. [166] classified normal breast epithelial cells from *BRCA1* mutation carriers into three types: basal, mature luminal, and luminal progenitor cells based on flow cytometry, and the Basal-like subtype of breast cancer showed high expression of the luminal progenitor cells gene signature, while the Luminal A and Luminal B subtypes exhibited high expression of the mature luminal cells gene signature, suggesting variability in the origins of different molecular subtypes of breast cancer.

Nguyen QH et al. [167] used scRNA-seq to obtain the transcriptome data of 25,790 primary human mammary epithelial cells from 7 individuals and found that normal mammary epithelial cells could be divided into three cell types: basal (*KRT14*^+^), luminal-1 (*KRT18*^+^*SLPI*^+^), luminal-2 (*KRT18*^+^*ANKRD30A*^+^). The gene scoring method revealed that the gene expression characteristics of the three normal breast epithelial cells of different breast cancer subtypes were different, demonstrating the possibility that different breast cancer subtypes had different origins. Other researchers have also identified 23 distinct subpopulations of normal breast epithelial cells, of which three mature luminal-like subpopulations and one luminal progenitor-like subpopulation may be the cell population that tend to develop into tumors [168]. Gray GK et al. [169] integrated single-cell transcriptome and proteome sequencing data to classify luminal progenitor cells into two distinct subtypes: basal-luminal (BL) and AV progenitor (AP). It was observed that the BL subtype exhibits an age-related accumulation pattern, with genomic characteristics resembling those of Basal-like breast cancer. Based on single-cell chromatin analysis, normal breast cells can also be classified into three subtypes: basal/myoepithelial (BM), luminal progenitor (LP), and mature luminal (ML) [170]. By comparing the scRNA-seq data of normal breast tissue and breast cancer, researchers found that the gene expression patterns of the Basal-like and Normal-like subtypes of breast cancer are more similar to the BM and LP subtypes of normal breast epithelium, while the Luminal A and Luminal B subtypes exhibit gene expression patterns similar to the ML subtype of normal breast epithelium [171].

Age is considered one of the significant risk factors for breast cancer [172]. To investigate the impact of aging on breast cancer, Li CM et al. [173] conducted scRNA-seq analysis on normal mammary tissues obtained from young and old mice. Their findings revealed a higher abundance of luminal progenitor cells in the mammary tissues of old mice, alterations in basal epithelial function, and an overall pro-inflammatory microenvironment within the tissue. These results provided insights into the underlying mechanisms linking aging to increased susceptibility to breast cancer. Pelissier Vatter FA et al. [174] conducted a single-cell proteome analysis of normal mammary epithelial cells from a cohort of 57 women aged 16 to 91. Their findings revealed that a specific subset of the apical luminal epithelium exhibited characteristics similar to Basal-like breast cancer, displaying enhanced cell adhesion and migration capabilities, progressively accumulating with age, thereby indicating an increased susceptibility to breast cancer.

Women with mutations in the *BRCA1* gene have an increased risk of developing breast cancer and a tendency to establish Basal-like breast cancer [175]. However, the underlying mechanisms remain unclear. By comparative analysis of scRNA-seq data from breast cancer tissues of *BRCA1* mutation carriers and non-carriers, as well as from normal breast tissues, the investigators found that Basal-like breast cancer and Luminal breast cancer may originate from Luminal progenitor cells and mature Luminal cells, respectively [176]. In addition to focusing on epithelial cells, the researchers discovered that pre-cancer-associated fibroblasts of individuals with *BRCA1* mutations produce tumorigenic factors that induce the accumulation of luminal progenitor cells and promote the develogtpment of breast cancer [177]. A recent study utilizing scRNA-seq data including over 800,000 normal breast tissue cells revealed that the immune cells of individuals with *BRCA1/BRCA2* mutations exhibit the expression of genes associated with immune exhaustion, suggesting the likelihood of immune evasion preceding tumor onset [178].

#### Exploring breast cancer heterogeneity with single-cell sequencing and spatial omics

Heterogeneity is one of the hallmarks of tumors, which often affects the oncogenesis, tumor progression, and treatment [179]. The causes of tumor heterogeneity remain unclear, and mainstream viewpoints include the tumor stem cell model [180] and the clonal evolution model [181]. In breast cancer, tumor heterogeneity is also very obvious [182]. The classical typing of breast cancer includes immunohistochemistry typing [2] and PAM50 typing [3]. With the deepening of research, multiple subtypes of a particular type of breast cancer have been classified, such as Lehmann BD et al. [183], who classified TNBC into six subtypes, Jiang YZ et al. [184], who classified TNBC into four subtypes, and Jin X et al. [185], who classified HR + /HER2- breast cancer into four subtypes. Different molecular subtypes of breast cancer have different therapeutic responses and clinical prognoses. Understanding the heterogeneity of breast cancer is of great significance for tumor prevention, clinical diagnosis and treatment [186]. SCS can describe the tumor landscape from the perspective of single cells, providing a new perspective for understanding the heterogeneity of tumor [187].

### Epithelial cells heterogeneity

Identifying the characteristics of normal breast tissue epithelial cells helps to understand the heterogeneity of breast cancer. As previously mentioned, SCS reveals that normal breast epithelial cells are composed of multiple cell types and exhibit considerable heterogeneity, which may explain the origin of different subtypes of breast cancer [167, 168].

In 2011, Navin N et al. [73] used SCS to study 100 tumor cells from two breast cancer patients and found that the tumor epithelium could be divided into three different clonal subgroups, and one single clonal subgroup will proliferate to form the primary tumor and spread to form metastasis. In 2018, Karaayvaz M et al. [188] analyzed scRNA-seq data of more than 1,500 cells from 6 TNBC breast cancer patients and found that tumor cells in different patients were composed of multiple different subgroups, and there were shared subgroups and self-specific subgroups among different patients. Moreover, the gene expression characteristics of a shared subgroup can also predict the prognosis of patients. In 2021, Wu SZ et al. [56] developed an intrinsic typing method (scSubtype) based on the scRNA-seq results of 26 patients with primary breast cancer (11 ER + , 5 HER2 + , and 10 TNBC). The scSubtype analysis revealed that in a single tumor sample, tumor cells consisted of multiple scSubtype molecular types that did not precisely match the histological type, reflecting the intratumoral heterogeneity of breast cancer. To investigate the impact of intratumoral heterogeneity on tumor prognosis, Xu L et al. [189] integrated 8 publicly available breast cancer scRNA-seq datasets to construct a comprehensive single-cell atlas comprising over 230,000 cells. They categorized tumor cells into ten distinct subpopulations through unsupervised and supervised clustering methods. They also developed a computational model called InteractPrint to assess the interactions between tumor cells and immune cells quantitatively. Notably, their findings demonstrated that the InteractPrint analysis of T cells could predict the therapeutic response of tumors to immune checkpoint blockade (ICB). The heterogeneity of breast cancer tumor cells are also reflected in spatial localization. When SCS was combined with spatial transcriptome sequencing, it was discovered that distinct subpopulations of tumor cells exhibited varying distribution patterns within the tumor [190].

### Microenvironment cells heterogeneity

Tumor is a complex disease. Besides the tumor cells themselves, the cellular and non-cellular components of the tumor microenvironment play a vital role in the process of tumorigenesis, metastasis, and recurrence [13]. The tumor microenvironment exhibits heterogeneity in composition and functional state, which arises from variations in tumor sites, intrinsic characteristics of tumor cells, tumor stages, and inter-individual differences among patients [191]. The cellular components of the tumor microenvironment mainly include immune cells such as T cells, B cells, monocytes, macrophages, and stromal cells such as fibroblasts, and the plasticity and heterogeneity of these cells influence tumor progression [192–196]. The application of SCS provides a more microscopic perspective for us to further analyze the cell heterogeneity in the microenvironment of different solid tumors [197, 198].

#### Immune cells

Savas P et al. [199] conducted SCS on 6311 T cells isolated from human breast cancer samples and identified 10 T cell clusters, revealing the significant heterogeneity of T cells. The authors focused on a subgroup of *CD8*^+^*CD103*^+^ T cells with tissue-resident memory T cell properties, which expressed multiple cytotoxic effector molecules and immune checkpoint molecules at high levels, and the gene expression characteristics of this cell subgroup were positively correlated with good prognosis in early TNBC patients. Azizi E et al. [200] constructed a scRNA-seq map of 47,016 *CD45*^+^ cells from tumor tissue samples, tumor metastatic lymph nodes, paired normal tissues, and peripheral blood from 8 breast cancer patients. By integrating all sequencing data, it was found that all immune cells could be divided into 38 T cell clusters, 27 myeloid cell clusters, 9 B cell clusters, and 9 NK cell clusters. Although tumor tissue, lymph nodes, normal breast tissue, and peripheral blood share some cell clusters, both cell types and cell abundance are further increased in tumors, demonstrating the complexity of the tumor microenvironment. Pal B et al. [201] used scRNA-seq to carefully compare the immune microenvironment characteristics of different breast cancer subtypes, revealing the cell diversity among different breast cancer subtypes. Wagner J et al. [202] drew a single-cell proteomic atlas of 144 tumor samples (including four breast cancer subtypes), 46 para-cancerous tissue samples, and 4 non-cancerous breast tissue samples. They made a detailed classification of epithelial cells, T cells, and myeloid cells. Different epithelial clusters, T cell clusters, and myeloid cell clusters are distributed differently in tumor tissue, paracancer tissue, and normal breast tissue, as well as among different breast cancer molecular subtypes, confirming the internal heterogeneity of different breast cancer molecular subtypes and suggesting the reasons for different responses to ICB. Programmed death ligand 1 (PD-L1) is predominantly expressed by tumor cells and is generally believed to exert immunosuppressive effects, leading to immune evasion by tumors [203]. Recent studies have indicated that tumor-associated macrophages (TAMs) can also exhibit high levels of PD-L1 expression [204]; however, the biological significance of these TAMs remains unclear. Utilizing scRNA-seq and spatial immunofluorescence staining techniques, Wang et al. [205] discovered that PD-L1^+^ TAMs possess immune-activating properties and are spatially adjacent to T cells. In vitro experiments further demonstrated that these TAMs can enhance the proliferation and cytotoxicity of *CD8*^+^ T cells. Moreover, in two independent breast cancer datasets, the presence of PD-L1^+^ TAMs was associated with a favorable prognosis.

#### Stromal cells

Cancer-associated fibroblasts (CAFs) are considered an important factor in promoting tumor progression and are highly heterogeneous cells [196]. In mouse models, SCS identified three types of CAFs: vascular CAFs (vCAFs), stromal CAFs (mCAFs), and developmental CAFs (dCAFs), all of which differ in their gene expression characteristics, origin, and spatial location. The above three types of CAFs also exist in human tumors, and the gene expression characteristics of vCAF and mCAF are independent predictors of metastasis of human breast cancer [206]. According to a previous study, CAFs in human breast cancer can be divided into four types: CAF-S1, CAF-S2, CAF-S3, and CAF-S4, and CAF-S1 is related to immunosuppressive microenvironment [207]. Cords et al. [208] conducted scRNA-seq on stromal cells of 14 breast cancer patients. Based on the sequencing results, nine types of CAFs and a type of pericytes were defined. Different subgroups of CAFs had different high-expression genes and functional states, which may guide clinical treatment in the future. To further investigate the role of various types of CAFs in tumor progression, Ye J et al. [209] identified a novel subgroup of CAFs called senescent CAFs (senCAFs) using publicly available scRNA-seq data. It was observed that senCAFs could suppress the toxicity of NK cells, thereby promoting tumor growth. Targeting senCAFs could potentially alleviate NK cell inhibition and restrict tumor growth. Kieffer Y et al. [210] individually sorted CAF-S1 from eight breast cancer samples by flow cytometry, identified eight distinct CAF-S1 subpopulations (ecm-myCAF/detox-iCAF/IL-iCAF/TGFβ-myCAF/wound-myCAF/IFNγ-iCAF/IFNαβ-myCAF/acto-myCAF) by using scRNA-seq. Croizer H et al. further investigated the function, plasticity, and spatial distribution of these subpopulations [211]. The results revealed that ecm-myCAF is associated with various immunosuppressive cells, such as *TREM2*^+^ macrophages, regulatory NK cells, and regulatory T cells. On the other hand, detox-iCAF is associated with immunoprotective *FOLR2*^+^ macrophages. In vitro and in vivo experiments demonstrated that tumor cells can induce the transformation of detox-iCAF into ecm-myCAF.

Growth disorders and abnormal functioning of blood vessels are indicative signs of tumors. Endothelial cells (ECs), the primary constituents of blood vessels, play a crucial role in regulating tumorigenesis and progression [212, 213]. ECs can be broadly categorized into ECs and vascular ECs, with the latter encompassing arterial, venous, and capillary ECs [214]. The advent of SCS has facilitated the analysis of EC’s plasticity and interactions with other cell types [215]. Geldhof V et al. [216] analyzed scRNA-seq data of 8433 breast cancer-associated ECs, revealing previously unreported potential interactions between these cells and immune cells that suggest possible immunomodulatory functions of ECs. Additionally, they identified a capillary EC subtype (Lipid processing endothelial cell: LIPEC) with high levels of expression of lipid metabolism genes. The retrospective analysis revealed that LIPEC was associated with a significantly improved prognosis in breast cancer patients treated with metformin.

### Application of single-cell sequencing in the study of breast *cancer* metastasis mechanism

Metastasis occurs in approximately 20–30% of breast cancer patients, and the 5-year overall survival rate of patients without distant metastasis can reach 80%, while the occurrence of distant metastasis can reduce this rate to 25% [217]. Almost all deaths of breast cancer patients are attributed to tumor metastasis [218], so the exploration of the mechanism of breast cancer metastasis is of great clinical value. The mechanism of breast cancer metastasis has been widely reported, involving many aspects [219–223]. SCS can provide new perspectives and ideas for studying metastatic mechanisms, which is conducive to revealing the differences in genetic, transcriptional, and metabolic features between primary and metastatic tumors.

An essential step in tumor metastasis is to break through the basement membrane and develop into invasive tumors [224]. To explore how breast ductal carcinoma in situ progresses into invasive ductal carcinoma, Casasent AK et al. [225] combined exon sequencing with topographic single cell sequencing to conduct an in-depth analysis of 1293 tumor cells from 10 breast cancer patients and trace the clonal evolution of their invasion process. Based on the sequencing data, the researchers reached three important conclusions: first, the genome of tumor cells evolved before the tumor cells broke through the basement membrane; second, the tumor subclones in breast ducts were all derived from single cells; third, tumor cell population that break through the basement membrane and migrate to neighboring tissues were derived from one or more clones.

The final step of tumor metastasis is colonization at distant sites, and the microenvironment at the sites of metastasis affects the ability of the tumors to metastasize to these sites [226]. Zou Y et al. [227] used scRNA-seq for the first time to analyze the microenvironment characteristics of brain and liver metastases in breast cancer. At the sites of metastasis, a variety of immunosuppressive cells such as *FOXP3*^+^ regulatory T (Treg) cells, *LAMP3*^+^ tolerogenic dendritic cells, *CCL18*^+^ M2-like macrophages, *RGS5*^+^ CAFs and *LGALS1*^+^ microglia cells increased significantly. The study also identified *KLF5* as a potent target for inhibiting breast cancer metastasis, with inhibitors that effectively inhibit the migration ability of breast cancer cell lines. The lymph node is the primary site of metastasis for a variety of solid tumors, including breast cancer [228] and tumors can metastasize to distant sites through lymph nodes [229], so understanding the mechanism of lymph node metastasis can provide a reference for clinical blocking of tumor lymph node metastasis. Lei PJ et al. [230] used a 4T1 mouse breast cancer model to study how tumor cells could metastasize and survive in lymph nodes. By conducting scRNA-seq analysis on paired samples of tumor primary and lymph node metastases, they discovered that the expression of genes controlling the expression of major histocompatibility complex (MHC) class II molecules were increased in tumor cells in lymph nodes, and the expression of costimulatory molecules was lacking, resulting in the decrease of CD4^+^ effector T cells and the increase of immunosuppressive Treg cells in lymph nodes. Experimental knockout of MHC-II gene expression in tumor cells resulted in decreased lymph node metastasis and decreased expansion of Treg cells in lymph nodes. Liu T et al. [231] studied the differences in tumor cells and tumor microenvironment between the primary tumor and lymph node metastases in human breast cancer and found that the activation, toxicity, and differentiation of T cells in tumor lymph node metastases were inhibited, while the antigen-presenting pathway of tumor cells was down-regulated. These findings explain why tumor cells can metastasize to lymph nodes. The most common site of distal metastasis of breast cancer is bone [232] and the mechanism of the high propensity of breast cancer to bone metastasis remains elusive. Wu Q et al. [233] first found that *SCUBE2* was highly expressed in luminal breast cancer and was related to bone metastasis and verified through experiments that this gene could promote bone metastasis of official breast cancer. To understand the causes of bone metastasis, the researchers conducted scRNA-seq on the bone metastasis niche of *SCUBE2*-positive tumor cells and bone metastasis niche of *SCUBE2*-negative tumor cells, and the results showed that osteoblasts were abundant in the former. Further studies have shown that *SCUBE2* can mediate the bone metastasis of tubular breast cancer by regulating the immunosuppressive osteoblast niche.

Tumor metabolism is also related to tumor metastasis, and metabolic plasticity is often enhanced during metastasis [234, 235]. How energy metabolism changes during breast cancer metastasis is not fully understood. Davis RT et al. [236] established a mouse transplanted tumor model of human xenogenic breast cancer and combined it with scRNA-seq to study the transcriptome differences between the primary tumor and metastatic tumor and found that the primary tumor and metastatic tumor showed high transcriptional heterogeneity. Pathway enrichment analysis showed that the oxidative phosphorylation pathway was significantly up-regulated in the metastatic tumor. In vitro experiments have also verified that inhibition of the oxidative phosphorylation pathway can lead to reduced metastasis, fully demonstrating the importance of the oxidative phosphorylation pathway in the process of metastasis. In addition, Liu YM et al. [237] used scRNA-seq and spatial transcriptome sequencing to analyze the paired samples of tumor primary sites and lymph node metastases from 4 breast cancer patients and also found that the energy metabolism transition from glycolysis to oxidative phosphorylation occurred in the early disseminated metastatic tumor cell population.

### Characteristics of drug resistance in breast cancer revealed by single-cell sequencing and spatial omics

Each subtype of breast cancer has unique biological characteristics and responds differently to drug treatments, necessitating individualized therapeutic approaches [172]. HR + HER2- breast cancer typically requires endocrine therapy, often combined with chemotherapy and targeted therapy; HER2 + breast cancer primarily relies on targeted therapies such as trastuzumab; and TNBC is mainly treated with chemotherapy and immunotherapy [4, 238]. However, the emergence of drug resistance has always posed a clinical challenge for these subtypes of breast cancer, although their resistance mechanisms are not entirely consistent.

#### HR + /HER2- breast cancer

HR + /HER2- breast cancer accounts for about 70% of all breast cancer cases. Endocrine therapy is one of the standard drug therapies for this breast cancer subtype [239, 240]. For some patients, CDK4/6 inhibitors, PARP inhibitors, and PI3K pathway inhibitors can be added according to the molecular characteristics of the tumors in order to enhance therapeutic efficacy [4, 172, 241]. Although endocrine therapy has achieved a significant effect, some patients still have endocrine therapy resistance, resulting in tumor metastasis or recurrence [242].

Brady SW et al. [243] followed up 4 patients with metastatic ER + breast cancer for many years and used whole genome sequencing, whole exome sequencing and single-cell DNA sequencing (scDNA-seq) to dynamically evaluate the subclonal evolution of patients' tumors during drug therapy. They found that drug-resistant tumor cells were present prior to chemotherapy, and chemotherapy led to the dominance of these cells, resulting in the development of chemotherapy resistance in the tumor. The mesenchymal and growth factor signaling pathways in these cells were upregulated, whereas the antigen presentation and TNF-α signaling pathways were downregulated. Griffiths JI et al. [244] collected tumor tissue samples from 60 postmenopausal women participating who received endocrine therapy and/or CDK4/6 inhibitors treatment at three-time points (at the beginning of treatment, day 14 of treatment, and at the end of treatment), and performed scRNA-seq and whole exon sequencing on these samples. The researchers found that the upregulation of ERBB4 signaling triggered the activation of the RTK signaling pathway to sustain estrogen signaling in drug-resistant tumor cells during endocrine monotherapy; during the combination therapy, the surviving resistant tumor cells exhibited accelerated loss of estrogen signaling and increased activation of growth factor receptor and JNK-MAPK signaling pathways, which facilitated the proliferation of these tumor cells. These results suggest that tumors can reduce their dependence on ER or CDK4/6 activation during endocrine therapy or combination therapy through a series of compensatory mechanisms, thereby circumventing inhibition by endocrine therapy and CDK4/6 inhibitors.

#### HER2 + breast cancer

HER2 + breast cancer accounts for approximately 20% of all breast cancers and is typically characterized by overexpression of HER2, which is significantly associated with poor prognosis. Due to the high expression of HER2, targeted HER2 agents have been one of the standard therapies for HER2 + breast cancer and have significantly improved the survival prognosis of patients with this type of breast cancer [245–248].

Trastuzumab, as the first humanized HER2 monoclonal antibody, has been widely used in clinical treatment. However, the emergence of drug resistance remains a significant challenge in clinical practice [249]. Du R et al. [250] used scRNA-seq to identify a critical cellular component of drug resistance out of trastuzumab, *PDPN*^+^CAF, that CAF induces tumor resistance by secreting indoleamine 2,3-dioxygenase 1 (IDO1) and tryptophan 2,3-dioxygenase 2 (TDO2), which inhibit the killing ability of NK cells against tumor cells. Drug targeting of IDO1 and TDO2 reversed the functional inhibition of NK cells by CAFs, thereby reducing tumor resistance to trastuzumab.

### TNBC

#### Chemotherapy

Compared to other breast cancer subtypes, TNBC lacks available clinical targets, exhibits a high risk of recurrence, and demonstrates a poor prognosis [251, 252]. Chemotherapy serves as the primary systemic treatment for patients with early and advanced TNBC, and many clinical studies have been conducted to explore strategies for improving the efficacy of chemotherapy in TNBC [253–256]. However, the emergence of chemotherapy resistance significantly contributes to clinical treatment failure and poor prognosis, resulting in a 5-year survival rate lower than that of non-TNBC breast cancer [257]. With further research advancements, the mechanism underlying TNBC chemotherapy resistance at single-cell resolution has gradually been elucidated.

Lee et al. [258] applied scRNA-seq to investigate metastatic breast cancer cell lines that were untreated, sensitive to paclitaxel chemotherapy, and resistant to paclitaxel chemotherapy, respectively. This study unveiled the dynamic cellular stress response mechanism under drug stimulation. The findings demonstrated the presence of various specific drug-resistant RNA variants in the drug-resistant cells, which were implicated in microtubule stabilization, cell adhesion, and cell surface signal transduction. Notably, the gene expression profile of the resistant cells resembled that of the untreated cells but exhibited a significant increase in expression abundance. Kim C et al. [259] conducted a longitudinal analysis of 20 patients with TNBC undergoing neoadjuvant chemotherapy (NAC) using scDNA-seq and scRNA-seq. Their findings revealed that the resistance genotype in TNBC was pre-existing and underwent adaptive selection during NAC. Following chemotherapy, TNBC patients exhibited adaptive genomic mutations, copy number aberrations, and drug-resistant phenotypes through transcriptional reprogramming. Therefore, the application of SCS aids in elucidating the mechanism of TNBC drug resistance during NAC, enabling clinicians to timely adjust the NAC regimen for optimal treatment outcomes in TNBC patients. By monitoring cell epigenomics and transcriptomics at single-cell resolution, Marsolier J et al. [260] found that the trimethylation modification of lysine 27 of histone chromatin 3 (H3K27me3) can regulate cell fate at the beginning of chemotherapy. Consumption of H3K27me3 enhanced the potential for chemotherapy tolerance in triple-negative breast cancer cell lines, and using inhibitors to block histone demethylation at this site reduced the number of resistant cells. Therefore, the application of SCS can help to explain the causes of chemotherapy resistance further and indicate the potential for further treatment of drug-resistant TNBC patients.

#### Immunotherapy

Tumor immunotherapy has revolutionized conventional cancer treatment by harnessing the power of the immune system to combat tumors [261–263]. Immunotherapy has demonstrated significant efficacy in TNBC due to its high immunogenicity and elevated expression levels of immune markers, such as PD-L1 and PD-1 (programmed death 1) [264, 265]. Currently, the primary focus of TNBC immunotherapy research lies in ICB which targets the PD-1/PD-L1 axis. However, although multiple clinical trials have demonstrated that ICB can benefit some patients with TNBC [266–268], the molecular characteristics of non-responders and the effective predictive markers for ICB treatment remain unclear.

Extensive research has been conducted to understand why only some patients respond to ICB and to identify markers that can predict the effectiveness of ICB treatment. Bassez A et al. [269] treated 29 newly diagnosed patients and 11 patients undergoing neoadjuvant chemotherapy with anti-PD-1 antibodies for about ten days. Paired pre- versus on-treatment biopsies were subjected to single-cell transcriptome, T cell receptor and proteome profiling. In pre-treatment biopsies, some cell types with potential contributions to immunotherapy outcomes were identified, particularly the cell subtypes associated with T cell clonal proliferation: immunoregulatory dendritic cells (*PD-L1*^+^), specialized macrophages (*CCR2*^+^ or *MMP9*^+^), and tumor cells expressing MHC-I/II were positively correlated with T cell expansion. In contrast, undifferentiated pro-effector/memory T cells (*TCF7*^+^, *GZMK*^+^) or inhibitory macrophages (*CX3CR1*^+^, *C3*^+^) were inversely associated with T cell expansion. Zhang Y et al. [270] analyzed the scRNA-seq results of 11 advanced TNBC patients who received paclitaxel monotherapy and 11 patients who received paclitaxel combined with atezolizumab before and after treatment. They found that *CD8*^+^*CXCL13*^+^ and *CD4*^+^*CXCL13*^+^ T cells could be used to predict the effective response to PD-L1 blockade, and B cells were the most prominent immune cells that predicted clinically effective responses to both treatments. In addition, paclitaxel also impaired the expansion of responsive immune cells induced by atezolizumab during combination chemotherapy. Dendritic cells are essential for activating the immune response and influencing the efficacy of tumor immunotherapy [271]. Wu SY et al. [272] delineated dendritic cells in triple-negative breast cancer (TNBC) at single-cell resolution. Within this study, a specific subpopulation of cells, termed "*CCL19*^+^ dendritic cells", exhibited a significant positive correlation with favourable responses to TNBC immunotherapy. Moreover, the levels of CCL19 content in both blood and tumors proved to be effective predictors of clinical benefit in patients undergoing anti-PD1 therapy.

The efficacy of tumor immunotherapy is closely associated with CAFs. Kieffer Y et al. [210] verified that ecm-myCAF and TGFβ-myCAF mediated resistance to ICB therapy in i melanoma and non-small cell lung cancer. It is worth exploring in the future whether these CAFs might also influence the efficacy of immunotherapy in breast cancer.

The spatial localization characteristics of cells within the tumor microenvironment have been shown to correlate with the efficacy of immunotherapy [273]. In TNBC, Wang XQ et al. [274] utilized imaging mass cytometry to obtain spatial protein expression profiles of tumor tissues from 279 patients during immunotherapy (before treatment, early treatment, and post treatment), highlighting different efficacy predictors at various treatment stages. Before treatment, the proliferation index of CD8^+^ TCF1^+^ T cells and MHCII^+^ tumor cells were the primary predictors of response, followed by interactions between tumor cells and immune cells (B cells and CD8^+^ GZMB^+^ T cells). During treatment, the characteristics of treatment-sensitive and resistant patients were the enrichment of CD8^+^ GZMB^+^ T cells and CD15^+^ tumor cells, respectively. Shiao SL et al. [275] sequenced tumor tissue samples from 50 patients who received pembrolizumab and radiotherapy (27 patients underwent spatial proteomics sequencing, and 34 patients underwent scRNA-seq). The researchers identified two types of patients who were sensitive to immunotherapy: one group had an abundance of immune cells before treatment, while the other group lacked immune cell infiltration before treatment but generated a strong immune response after immunotherapy and radiotherapy. Significant interactions between effector T cells and antigen-presenting macrophages characterized this strong immune response.

Adoptive cell therapy (ACT) is an immunotherapy method in which the immune cells of the patient are modified in vitro and then transfused back into the patient to enhance anti-tumor immunity, including chimeric antigen receptor T cells (CAR-T) therapy, chimeric antigen receptor-engineered natural killing cells (CAR-NK) therapy and dendritic cells vaccines, among others [276]. ACT has shown promising results in the treatment of certain types of tumors [277–279]. CAR-T therapy is progressively being incorporated into clinical trials for validation of its effectiveness in TNBC [280]. Although there is presently no SCS study conducted in cohorts of breast cancer patients undergoing ACT treatment, SCS has exhibited a crucial role in elucidating the mechanism of ACT and identifying effective biomarkers in other tumors [281–284]. In the future, as ACT becomes more established in breast cancer treatment, it is believed that SCS can further optimize its application effectiveness.

## Conclusions

The application of SCS and spatial omics has brought about revolutionary changes in breast cancer research. Firstly, understanding the origin of breast cancer is crucial for the prevention, early diagnosis, and treatment of this common cancer. SCS and spatial omics have unveiled a novel perspective on the origin of breast cancer. Secondly, given the high heterogeneity of breast cancer, precise individualized treatment and stratified treatment are of paramount importance. Through analysis at the single cell level, we can gain a more comprehensive and precise understanding of breast cancer heterogeneity. This holds significant implications for personalized treatment, as the cellular heterogeneity of breast cancer may lead to different treatment responses and the development of drug resistance in different patients and at various stages of the disease.

Additionally, tumor metastasis is one of the main reasons leading to poor prognosis for patients. At the resolution of a single cell, we can track the microscopic changes of cells during metastasis, deeply understand the mechanisms that trigger tumor metastasis, and provide guidance for reducing tumor metastasis and improving patient prognosis. However, SCS and spatial omics have some limitations, such as high sequencing costs, sequencing technology noise, and complex data analysis processes. When SCS is not feasible due to various constraints, publicly available single-cell and spatial omics databases can be an alternative resource (Table 1). Although no dedicated database for breast cancer exists, relevant data can be accessed through numerous published literature (Table 2).

**Table 2:** Summary of the publicly published single-cell and spatial omics datasets for normal human breast and breast cancer

Source	Description	Method	Availability
Nguyen QH et al. [167]	7 samples from healthy breast	scRNA-seq	GSE113197
Bhat-Nakshatri P et al. [168]	5 samples from healthy breast	scRNA-seq	Correspondence with authors
Gray GK et al. [169]	16 samples from healthy breast	scRNA-seq	GSE180878
Pelissier Vatter FA et al. [174]	50 samples from healthy breast	Single-cell mass cytometry	https://doi.org/10.17632/j7mrbgt3hh.1
HU L et al. [176]	Breast cancer tissues and adjacent or prophylactic normal breast tissues from BRCA1 germline variant carriers (n = 4) and noncarriers (n = 3)	scRNA-seq	Correspondence with authors
Nee K et al. [177]	BRCA1 germline variant carriers (n = 11) and noncarriers (n = 11)	scRNA-seq	GSE174588
Reed AD et al. [178]	2.1 million cells from normal breast tissue from 286 individuals	scRNA-seq	E-MTAB-13664
Navin N et al. [73]	2 breast cancer samples	scDNA-seq	SRA018951
Karaayvaz M et al. [188]	Breast cancer samples (6 TNBC)	scRNA-seq	GSE118390
Wu SZ et al. [56]	Breast cancer samples (11 ER + , 5 HER2 + , 10 TNBC)	scRNA-seq	GSE176078
Liu SQ et al. [190]	Breast cancer samples (2 HR + HER2 +)	scRNA-seq spatial transcriptomics	Correspondence with authors
Savas P et al. [199]	Breast cancer samples (2 TNBC)	scRNA-seq	GSE110686
Azizi E et al. [200]	Breast cancer samples (5 ER + , 1 HER2 + , 2 TNBC) and matched normal breast tissue, peripheral blood, and lymph node	scRNA-seq	GSE114727 GSE114725
Pal B et al. [201]	69 samples encompassing normal breast(n = 24), preneoplastic BRCA1 + /– tissue(n = 4), breast cancer samples (20 ER + , 6 HER2 + , 8 TNBC), and matched lymph node(n = 7)	scRNA-seq	GSE161529
Wagner J et al. [202]	144 breast cancer samples (54 luminal A, 71 luminal B, 6 luminal B-HER2 + , 1 HER2 + , 6 TNBC) and 50 non-tumor tissue samples	Single-cell mass cytometry	https://data.mendeley.com/datasets/gb83sywsjc/1
Wang L et al. [205]	Breast cancer samples (5 ER +)	scRNA-seq	GSE248288
Cords L et al. [208]	14 breast cancer samples (9 ER + , 1 ER + HER2 + , 4 TNBC)	scRNA-seq	https://zenodo.org/records/7540604
Kieffer Y et al. [210]	8 breast cancer samples	scRNA-seq	EGAS00001004030
Casasent AK et al. [225]	10 breast cancer samples (4 ER + , 1 ER + HER2 + , 5 TNBC)	TSCS	SRP116771
Zou Y et al. [227]	6 liver and brain metastases of breast cancer	scRNA-seq	Correspondence with authors
Liu T et al. [231]	8 breast cancer samples (5 ER + , 3 HER2 +) and matched lymph node metastases	scRNA-seq spatial transcriptomics	GSE167036 GSE190811
Liu YM et al. [237]	4 breast cancer samples (1 ER + , 2 ER + HER2 + , 1 HER2 +) and matched lymph node metastases	scRNA-seq spatial transcriptomics	GSE225600
Brady SW et al. [243]	4 metastatic ER + breast tumors	scDNA-seq	EGAS00001002436
Griffiths JI et al. [244]	34 patients with endocrine or CD4/6 inhibitor resistant ER + breast cancer	scRNA-seq	GSE158724
Du R et al. [250]	6 HER2 + breast cancer samples (3 trastuzumab responders and 3 non-responders)	scRNA-seq	Correspondence with authors
Kim C et al. [259	4 breast cancer samples (4 TNBC)	scRNA-seq scDNA-seq	SRP114962
Bassez A et al. [269]	Treatment-naive patients treated with pembrolizumab (n = 29) and patients treated with neoadjuvant chemotherapy before receiving pembrolizumab (n = 11)	scRNA-seq scTCR-seq	EGAS00001004809
Zhang Y et al. [270]	22 TNBC patients treated with paclitaxel or its combination with atezolizumab	scRNA-seq scATAC-seq	GSE169246
Wu SY et al. [272]	31 TNBC patients treated with pembrolizumab	scRNA-seq	Correspondence with authors
Wang XQ et al. [274]	279 TNBC patients treated with pembrolizumab	Imaging mass cytometry	https://zenodo.org/records/7990870
Shiao SL et al. [275]	50 TNBC patients treated with pembrolizumab	scRNA-seq scTCR-seq scBCR-seq CODEX	GSE246613 https://doi.org/10.5281/zenodo.10045066

*Abbreviations*: *ER* estrogen receptor, *HR* hormone receptor, *HER2* human epidermal growth factor receptor 2, *TNBC* triple-negative breast cancer, *snRNA-seq* single-nucleus RNA sequencing, *scATAC-seq* single-cell assay for transposase-accessible chromatin using sequencing, *scTCR-seq* single-cell T cell receptor sequencing, *scBCR-seq* single-cell B cell receptor sequencing, *TSCS* topographic single cell sequencing, *CODEX* co-detection by indexing

Future research endeavors should prioritize the development of streamlined and more efficient sequencing protocols and data analysis methodologies while concurrently reducing the associated costs. This will facilitate broader adoption and dissemination of SCS and spatial omics. In addition, how to effectively use these advanced sequencing technologies is also the focus of our future research. For breast cancer, these techniques should be employed to gain a better understanding of gene expression variability and tumor microenvironment complexity in order to identify crucial therapeutic targets. The analysis of the immune microenvironment in milk cancer, for instance, may facilitate the identification of specific immunotherapy strategies and enhance the efficacy of existing treatments. Additionally, in order to advance the personalized treatment of breast cancer patients, it is crucial to leverage SCS and spatial omics technologies for the identification of novel biomarkers.

Overall, the widespread use of SCS and spatial omics in breast cancer research allows us to gain insight into tumor biology and individualized therapy. By analyzing the individual properties of tumors, we can take a critical step toward more precise and effective breast cancer treatment.

## Data Availability

No datasets were generated or analysed during the current study.

## Abbreviations

CAFs: Cancer-associated fibroblasts
dCAFs: Developmental CAFs
CODEX: Co-detection by indexing
DR-seq: GDNA-mRNA sequencing
ECs: Endothelial cells
ER: Estrogen receptor
HR: Hormone receptor
HER2: Human epidermal growth factor receptor 2
IHC: Immunohistochemistry
ICB: Immune checkpoint blockade
LIPEC: Lipid processing endothelial cell
MHC: Major histocompatibility complex
mCAFs: Stromal CAFs
PR: Progesterone receptor
PD-L1: Programmed death ligand 1
PD-1: Programmed death 1
SCS: Single-cell sequencing
scRNA-seq: Single-cell RNA sequencing
scDNA-seq: Single-cell DNA sequencing
snRNA-seq: Single-nucleus RNA sequencing
scATAC-seq: Single-cell assay for transposase-accessible chromatin using sequencing scTCR-seqSingle-cell T cell receptor sequencing
scBCR-seq: Single-cell B cell receptor sequencing
senCAFs: Senescent CAFs
SNARE-seq: Single-nucleus chromatin accessibility and mRNA expression sequencing
TAMs: Tumor-associated macrophages
TNBC: Triple-negative breast cancer
vCAFs: Vascular CAFs
